# Supplementary material for: MetaRibo-Seq measures translation in microbiomes
Source: Nat Commun. 2020 Jun 29;11:3268. doi: 10.1038/s41467-020-17081-z (PMC7324362; doi:10.1038/s41467-020-17081-z)
Supplement: Supplementary file 10 — Supplementary Data 7 [file 41467_2020_17081_MOESM10_ESM.zip › File2/Confidence_High_Taxonomy/307371_out.krona.html]

Javascript must be enabled to view this page.

members
magnitude
magnitudeUnassigned
count
unassigned
taxon
rank

307371\_out

9

superkingdom
2
9

9
976
phylum

200643
class
9

9
order
171549

815
family
9

9
genus
816


SRS014313\_contig\_number\_contig-100\_434.50917SRS018836\_contig\_number\_14498SRS019445\_contig\_number\_19464SRS058723\_contig\_number\_2044SRS077335\_contig\_number\_1480SRS104975\_contig\_number\_4181SRS143895\_contig\_number\_contig-100\_265.263672SRS147346\_contig\_number\_45700SRS148196\_contig\_number\_37446
9
820
species
